# Comparing machine learning and deep learning regression frameworks for accurate prediction of dielectrophoretic force

**DOI:** 10.1038/s41598-022-16114-5

**Published:** 2022-07-13

**Authors:** Sunday Ajala, Harikrishnan Muraleedharan Jalajamony, Midhun Nair, Pradeep Marimuthu, Renny Edwin Fernandez

**Affiliations:** 1 Department of Engineering, Norfolk State University, Norfolk, USA; 2 APJ Abdul Kalam Technological University, Thiruvananthapuram, India; 3 Rajeev Gandhi College of Engineering and Technology, Puducherry, India

**Keywords:** Electrical and electronic engineering, Nanoscale biophysics

## Abstract

An intelligent sensing framework using Machine Learning (ML) and Deep Learning (DL) architectures to precisely quantify dielectrophoretic force invoked on microparticles in a textile electrode-based DEP sensing device is reported. The prediction accuracy and generalization ability of the framework was validated using experimental results. Images of pearl chain alignment at varying input voltages were used to build deep regression models using modified ML and CNN architectures that can correlate pearl chain alignment patterns of Saccharomyces cerevisiae(yeast) cells and polystyrene microbeads to DEP force. Various ML models such as K-Nearest Neighbor, Support Vector Machine, Random Forest, Neural Networks, and Linear Regression along with DL models such as Convolutional Neural Network (CNN) architectures of AlexNet, ResNet-50, MobileNetV2, and GoogLeNet have been analyzed in order to build an effective regression framework to estimate the force induced on yeast cells and microbeads. The efficiencies of the models were evaluated using Mean Absolute Error, Mean Absolute Relative, Mean Squared Error, R-squared, and Root Mean Square Error (RMSE) as evaluation metrics. ResNet-50 with RMSPROP gave the best performance, with a validation RMSE of 0.0918 on yeast cells while AlexNet with ADAM optimizer gave the best performance, with a validation RMSE of 0.1745 on microbeads. This provides a baseline for further studies in the application of deep learning in DEP aided Lab-on-Chip devices.

## Introduction

Tools like DL and ML are integral part of artificial intelligence^1–3^. ML for image analysis typically involves extraction of important features from an image and training a machine learning model^4^. Machine learning can be highly efficient when the extracted features distinctly represent a particular image. Images need to be converted into feature vectors and train a model^4–6^. are examples of approaches where machine learning has been used to predict the presence, absence or possibility of an occurrence in images. However, extraction of significant features from complex images is intricate. Alternatively, deep learning does not depend on an input feature. Rather, DL models identifies significant features from processed images and classifies them based on the identified features. Feature maps extracted through deep learning from computed tomography (CT), magnetic resonance imaging (MRI), positron emission tomography (PET), mammography, ultrasound, and histopathology, provide valuable information ^4,7,8^. In cellular biology, DL-based approaches are primarily adopted to detect changes in cell morphology and correlate them to the mechanisms governing drug response^7,8^. Images of brain, prostate, retina, lungs are often combined with deep learning algorithms to predict medical conditions. U-Net, ResNet, and VGG are the most frequently used Convolutional neural network-derived networks for medical image segmentation and classification tasks. Recently, transfer learning and GAN-derived networks were widely applied in COVID-19 studies. Although, DL training involves intense data processing and long training time, it gives accurate predictions when used with high performance GPU and labelled data. In this study, we have designed models using both machine learning and deep-learning approaches to estimate the magnitude of dielectrophoretic force from microparticle alignment in a point-of-care device.

### Significance

Application of DEP in point-of care sensing devices demands two important requirements—(1) Low voltage (< 10 Voltage) physical device (2) an intelligent system that can correlate microparticle pearl-chain formation into dielectrophoretic force.

The dielectrophoretic force (${F}_{\mathrm{DEP}}$) invoked on a microparticle can be directly correlated to its dielectric property changes (Eq. 1). The DEP force is also proportional to the electric field intensity, particle dimension and medium conductivity^9–12^. Practically, particle alignment with respect to the electrodes, at a particular voltage and frequency is taken as an indicator of DEP force. Although particle alignment differs from experiment to experiment, some of the features of the particle aggregates are dominant and unique. ${F}_{\mathrm{DEP}}$ exerted on microparticles drive them into pearl chain assemblies which eventually will be aligned along the electric field^13,14^. For instance, the number of particles in a pearl chain at an applied voltage has been found relatively constant. The pattern has been confirmed by several researchers in the past. In an experiment with 5 µm PS beads^15^, pearl chains with 10–12 beads where formed for an applied potential of 15 V_pp_ at 200 kHz. Likewise,10 µm PS beads formed pearl chains with 7–12 beads at 20 V_pp_ at 20 MHz in a low conductivity buffer (1.8 × 10^−4^ S/m)^13^. In^16^ negative DEP of the PS beads was observed when a voltage of 3.8 V_pp_ of 480 kHz frequency was applied, forming 6–7 beads-long pearl chain. Similar studies on yeast cells have been reported when voltage (3.7 V_pp_) at a field frequency of 580 kHz exhibited positive DEP, the number of particles aggregated were found to be related to the applied voltage^16,17^.

Most of the studies have been reported on Lab-on Chip devices with precise geometrical features. Electrode aided DEP systems are mainly based on planar electrodes^18–21^ which require voltages between 10–20 V. Electrodeless DEP (i-DEP) systems, operated at voltages in the 20–100 V range, are used for studying the dielectric variations of biological cells and polymer beads^22,23^. Advances in nanofabrication and nanotechnology have resulted in several DEP aided Lab-on-a-chip (LOC) systems that are used for microparticle separation^24–26^, identification^21,27^, concentration^28^. Several studies have reported dielectric characterization of biological particles such as viruses, bacteria, fungi, protozoa, proteins, lipids, and DNA^21,29–32^ using DEP aided LOC systems. DEP aided immunological sensing is advantageous as they increase the local concentration of target particles thereby enhancing the overall device sensitivity and response time^33^. DEP aided LOC has been utilized in conjunction with scanning electron microscopy (SEM) to capture and immobilize viable cells in the SEM region without the use of a chemical surface modification^34^.

The requirement to precisely estimate ${F}_{\mathrm{DEP}}$ induced on particles is critical in the development of efficient microfluidic systems. ${F}_{\mathrm{DEP}}$ estimation is a necessity in DEP aided dielectric characterization systems used in clinical diagnostics of biological cells^32^. Patterns of microparticle aggregation is a direct indicator of ${F}_{\mathrm{DEP}}$ and can be correlated to the dielectric properties of microparticles including biological cells^21,22,31,35,36^. However, due to drag force phenomenon, the yield of LOC devices is extremely low at higher flow rates^37^. To circumvent this constraint, the ${F}_{\mathrm{DEP}}$ induced on the particles should be increased substantially^31,32,38^ resulting in a need for 3D microelectrodes. The cost of creating LOC systems with 3D electrodes involve nanofabrication techniques that are prohibitively high. We have developed an alternative by using textile electrodes that can invoke high ${F}_{\mathrm{DEP}}$ due to its high surface area.

In our previous work^12,17^, we have reported the use of textile electrode-based DEP device in conjunction with deep learning to predict the ${F}_{\mathrm{DEP}}$ induced on PS microbeads where we cast the ${F}_{\mathrm{DEP}}$ estimation task as a classification problem. Convolutional Neural Networks (CNNs) classifiers were used to estimate the applied DEP voltage from the pearl chain alignment of the microbeads. This was done to tackle the limitations in the existing ${F}_{\mathrm{DEP}}$ estimation techniques such as the equivalent dipole (EDM)^39,40^, Maxwell stress tensor (MST)^41–44^, iterative dipole moment (IDM)^9,40,45,46^ or velocity tracking method. We had used a classification-oriented CNN method in training the CNN models, which though gave excellent training results, performed poorly on testing with adversarial samples^12,17^. Also, classification paradigm may be inadequate for accurate ${F}_{\mathrm{DEP}}$ computation as the spacing between distinct voltages is crucial. This can only be modeled accurately using a regression framework.

In this work we have treated the ${F}_{\mathrm{DEP}}$ estimation task as a regression problem^47–49^ and have used ML and DL models for analyses. Regression models in computer vision cover a wide range of scenarios, including head-position estimation^50^, face landmark recognition^51^, and age estimation^52,53^. Regression is commonly used to tackle problems requiring the prediction of continuous values. CNN-based deep regression approach has been adopted for cell counting using micrographs^48,54^. In such situations, the softmax layer is often replaced with a fully connected regression layer with linear or sigmoid activations. Also, the softmax loss which is widely used for classification tasks is replaced with Euclidean loss for regression^48^.

In this research, we have explored ML algorithms like Random Forest, KNN, MLP, SVM, and Linear Regression to determine the ${F}_{\mathrm{DEP}}$ experienced by polystyrene beads and yeast cells in a low conductivity buffer. Because of the ML algorithms especially on out-of-sample and adversarial samples, we further experimented extensively with DL architectures which has the capacity to learn complex data representations with greater accuracy. Using ML approach as a bench mark we explain how deep learning may be used to accurately determine the ${F}_{\mathrm{DEP}}$. AlexNet, ResNet-50, MobileNetV2, and GoogLeNet are the four pre-trained CNN architectures in this study that were modified into deep regression CNN architectures. We trained the models to predict applied voltages from micrographs of polystyrene and yeast cell pearl chains generated during dielectrophoresis using transfer learning. The abbreviations and words listed in Table 1 will be used throughout the rest of the paper.

**Table 1:** A list of abbreviations and terms used in the paper.

Abbreviation	Meaning
ML	Machine learning
DL	Deep learning
DEP	Dielectrophoretic
CNN	Convolutional neural networks
PS	Polystyrene
KNN	K-nearest neighbor
SVM	Support vector machine
MAE	Mean absolute error
MRE	Mean absolute relative
MSE	Mean squared error
RMSE	Root mean square error
CT	Computed tomography
MRI	Magnetic resonance imaging
PET	Positron emission tomography
i-DEP	Electrodeless DEP
LOC	Lab-on chip
SEM	Scanning electron microscopy
EDM	Equivalent dipole
MST	Maxwell stress tensor
IDM	Iterative dipole moment
AC	Alternating current
ID	Inner diameter
OD	Outer diameter
ReLU	Rectified linear unit
ADAM	Adaptive moments
RMSProp	Root Mean square propagation
SGDM	Stochastic gradient descent with momentum
AdaGrad	Adaptive gradient
Li	Pearl chain lengths

## Materials and methods

### Dielectrophoretic force estimation theory

The interaction of a non-uniform electric field with a dipole is known as dielectrophoretic force ${F}_{\mathrm{DEP}}$.To improve the approximation of the ${F}_{\mathrm{DEP}}$ exerted on the particles in terms of the voltage applied, a more tangible and straightforward model is required to advance DEP aided sensing systems. The ${F}_{\mathrm{DEP}}$ when the particle is significantly smaller than the non-uniformities in the electric field is given in^17^ as:

1 $${F}_{\mathrm{DEP}}=2\pi {a}^{3}{\varepsilon }_{m}Re\left({f}_{cm}\right)\nabla {E}_{rms}^{2}$$

where $a$ is the radius of spherical microparticles in a medium, under an alternating current (AC) field ${E}_{rms}$; ${F}_{\mathrm{DEP}}$ depends on the product of the localized field with its gradient ($\nabla {E}_{rms}^{2}$) and the frequency-dependent complex dielectric contrast of the particle versus the medium, as given by real-part of the Clausius–Mossoti factor $Re\left({f}_{cm}\right)$;

2 $${f}_{cm}=Re\left(\frac{{\varepsilon }_{p}^{*}-{\varepsilon }_{m}^{*}}{{\varepsilon }_{p}^{*}+{2\varepsilon }_{m}^{*}}\right)$$

where ${\varepsilon }_{p}^{*}$ and ${\varepsilon }_{m}^{*}$ are the complex permittivities of the microparticles and the medium, respectively; and given as:

3 $${\varepsilon }_{p}^{*}={\varepsilon }_{p}-i\frac{{\sigma }_{p}}{\omega }$$

4 $${\varepsilon }_{m}^{*}={\varepsilon }_{m}-i\frac{{\sigma }_{m}}{\omega }$$

where $i= \sqrt{-1}$ and $\omega$ is the angular frequency of the applied AC field.

The DEP fingerprints, or dielectric properties, of a particle in a certain media can be determined by altering the AC signal frequency. Particles can be manipulated once their DEP fingerprints have been discovered. For particle chains, ${F}_{\mathrm{DEP}}$ can be theoretically calculated^55^ using multipole re-expansion and the method of images. ${F}_{DEP}$ on a particle chain is highly dependent on the angle between the applied field and the chain. The maximum attractive and repulsive forces in a chain grow significantly with the number of particles in the chain, but when the number of particles is large enough, they reach saturation. This ${F}_{DEP}$ was analytically calculated and given as^17^:

5 $${F}_{DEP}={\varepsilon }_{E}{\varepsilon }_{0}E{E}_{n}-\frac{1}{2}{\varepsilon }_{E}{\varepsilon }_{0}{E}^{2}n$$

where ${\varepsilon }_{E}$ the relative permittivity of the medium, E is the electric field on the microparticle surface, ${E}_{n}$ is the normal component of **E**, and **n** is the unit normal vector on the surface. However, theoretical estimate of ${F}_{DEP}$ from micrographs is problematic due to discrepancies in the thread structure. At the microscopic level, the orientation of textile strands differs greatly. The calculated force becomes ambiguous as a result.

The applied voltages are predicted by examining the patterns of pearl chain orientation. From the micrographs collected, the deep learning regression algorithms predicted the applied voltage on particles. The force on a chain of spherical dielectric particles in a dielectric fluid is proportional to the number of particles as well as its orientation to the electric field, according to various studies^55,56^. As a result, a direct link between the applied voltage and the pearl chain formation has been established.

### Problem formulation

Let us assume that the $j-th$ image is defined in an input space ${x}_{j}\in X$, and there is an output space ${y}_{i}\in Y = \{{u}_{1}, {u}_{2}, \cdot  \cdot  \cdot  , {u}_{k}\}$ with sorted ranks ${u}_{k}\gg {u}_{k-1}\gg \cdot  \cdot  \cdot  \gg {u}_{1}$. The symbol $\gg$ represents how different rankings are ordered. Given a training dataset $\upchi ={\{{x}_{i}, {y}_{i}\}}_{i=1}^{N}$, the goal of regression is to create a mapping from pearl chain images to ranks $g(.): X\to Y$ such that the risk functional $R(g)$ is minimized using a specified cost $c: X\times Y\to R$. The cost matrix $C$ is used to calculate the difference in cost between predicted and ground-truth ranks in this research^53^. $C$ is a $K\times K$ matrix with ${C}_{y,u}$ denoting the cost of predicting a sample $(x, y)$ with rank $u$. Normally,

when $u\ne y$, ${C}_{y,u}>0$ and ${C}_{y,y}=0$ are assumed. For general regression issues, the absolute cost matrix, which is defined as ${C}_{y,u}= |y-u|$, is a frequent choice. When applying regression techniques to ${F}_{\mathrm{DEP}}$ estimation, each voltage is treated as a rank.

### Machine learning aided pearl chain detection from DEP micrographs

**Figure 1:**
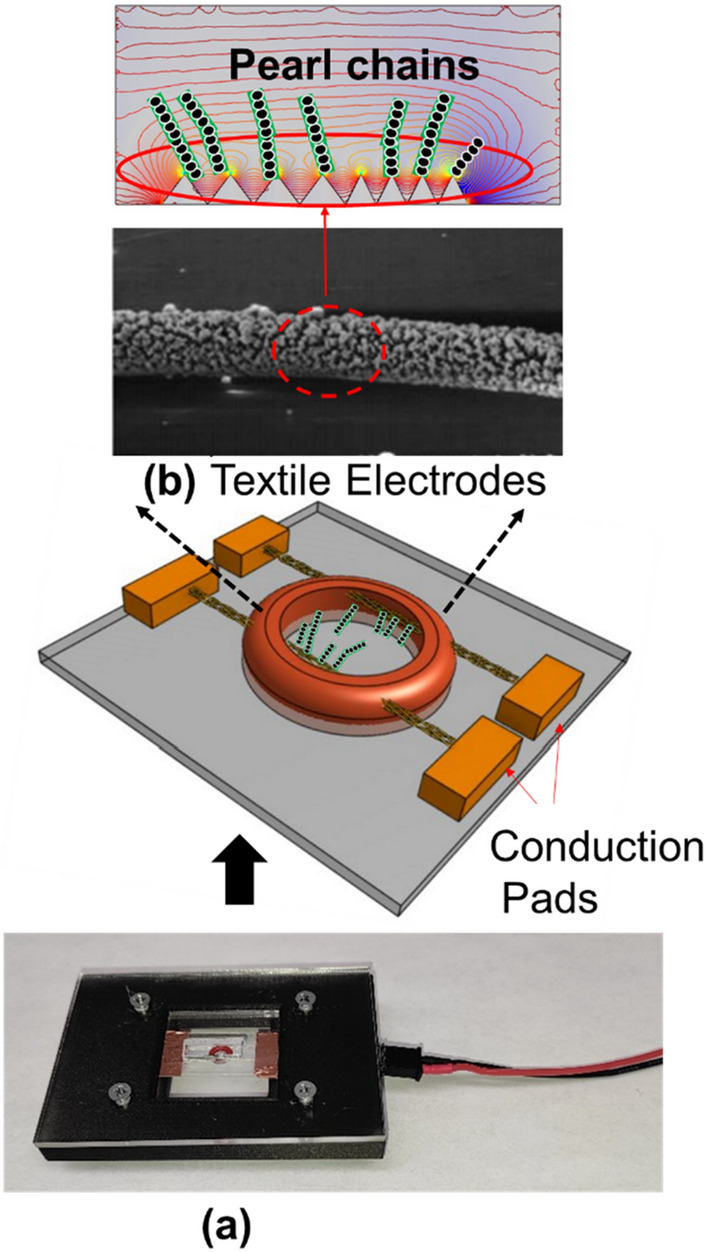
(**a**) Textile electrode-based DEP device with the connection base (**b**) SEM micrograph of textile electrodes.

The DEP framework device (Fig. 1) comprises flexible textile electrodes sewn through a silicon O-ring (ID: 1 mm, OD: 3 mm). The textile electrodes are silver-coated conductive string, 82% nylon, and 18% silver. This structure was mounted on a 1 × 1 inch glass slide. Strings were secured using copper tape, which acted as an electrical contact. Tests were performed by introducing 10 μL of fluid into the O-ring chamber. A 3D printed custom microscope stage encloses the whole gadget for recording pictures. The pearl chain formations were recorded at different voltages at a fixed frequency of 200 kHz (Fig. 1b). During our dielectrophoresis experiments with yeast cells and 10–20 µm sized PS microbeads using this setup, 200 micrographs were collected at each voltage level from 1–10 V for yeast cells and polystyrene microbeads, making a sum of 4000 images.

Yeast cells (Saccharomyces cerevisiae) are grown in an incubator at 30 °C. The growth medium yeast extract peptone dextrose consisted of 20 g/l peptone, 10 g/l yeast extract, and 20 g/l dextrose dissolved in deionized (DI) water. The cells were collected at the stationary growth phase after 1 day of culture in shaking incubator, and they were harvested by centrifugation for 2 min at 3000 rpm and re-suspended in measurement buffers. Plain polystyrene (PS) beads (10, 20 µm) were purchased from Spherotech, Inc., USA. PS beads are charge neutral and are hydrophobic. There was no surface functionalization used. The buffer did not include any surfactant.

Low conductivity buffer: All the microparticles were suspended in an isotonic buffer consisting of 200 mM sucrose, 16 mM glucose, 1 MCaCl_2_, and 5 mM Na_2_HPO_4_ in DI water (pH 7.4) for the experiments.

#### Feature extraction for machine learning based regression analysis

**Figure 2:**
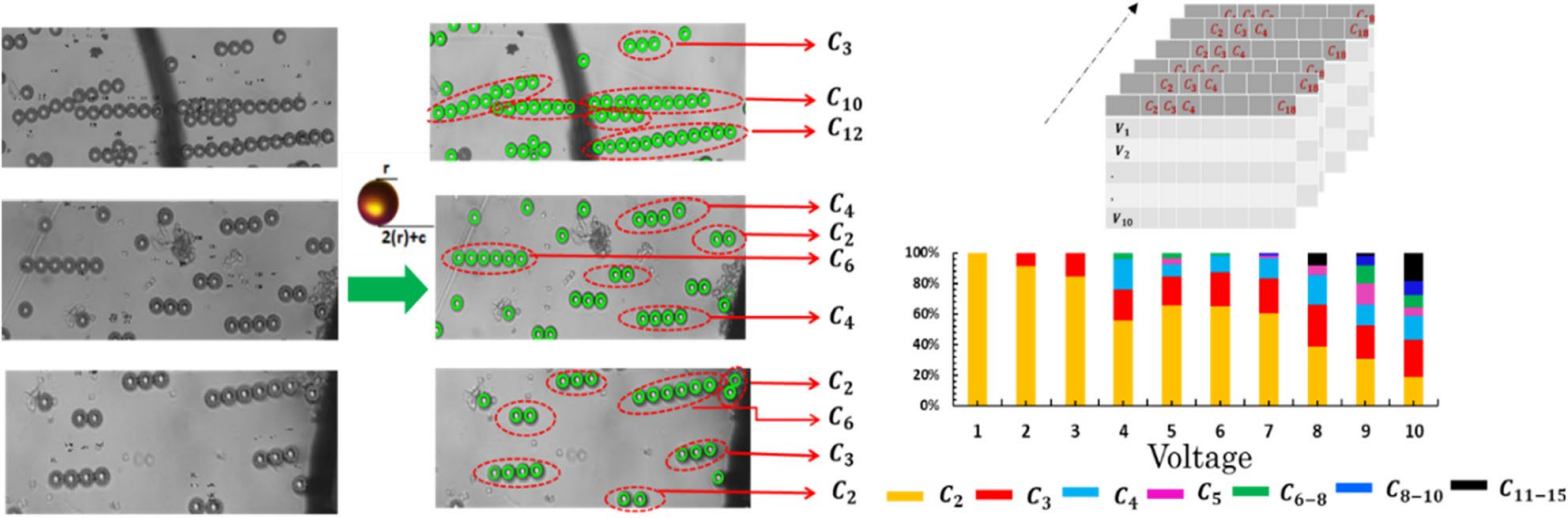
Pearl chain analysis using a Template-Based Matching algorithm and particle coordinate search algorithm. A template image is shifted across the DEP micrographs by an offset (x, y) using the origins of the two images as reference points. Pearl chain lengths (Li) is determined using a particle coordinate search algorithm.

We designed a template matching algorithm for object detection using $OpenCV$ (algorithm I) to extract the total number of pearl chains in an image, count each pearl chain and map them into a matrix that represents these features. (Fig. 2). Pearl chains are identified within the image using reference shapes which are the cropped images of individual microparticles which is the recognition template. The image dimensions of the template image are also extracted i.e. height, width, to calculate the radius of the microparticle. The radius of a sample pearl in unit of pixels is calculated as $r = (l + b)/4$ where $l$ is the length and $b$ is the breadth of the template image of microparticle. In the formula $c$ is a constant which is fixed at $1/4$ of $r$. The value of $c$ can be corrected until the output data set includes the data of undetected pearl chains.

The input image is represented as $I(x, y)$, with $(x, y)$ denoting the pixel coordinates. $T\left({x}^{^{\prime}},{y}^{^{\prime}}\right)$ denotes the coordinates of each pixel in the template. Template-Based Matching is done by simply moving the center (or the origin) of the template $T\left({x}^{^{\prime}},{y}^{^{\prime}}\right)$ over each $(x, y)$ point in the input image and calculate the sum of products between the coefficients in $I(x, y)$ and $T\left({x}^{^{\prime}},{y}^{^{\prime}}\right)$, over the whole area spanned by the template. As all possible positions of the template with respect to the input image are searched, the position with the highest score is the best position. In the OpenCV implementation, for each location of T over I, we store the cross correlation metric $(TM\_CCORR\_NORMED)$ in the result matrix R. The cross correlation metric $(TM\_CCORR\_NORMED)$ used is depicted mathematically as $R(x, y)$ in Eq. 6. Each location $(x, y)$ in R contains the match or cross correlation score, which is the result of sliding the patch with a metric $TM\_CCORR\_NORMED$. The brightest locations indicate the highest matches.

6 $$R\left(x, y\right)=\frac{{\sum }_{{x}^{^{\prime}},{y}^{^{\prime}}}T\left({x}^{^{\prime}},{y}^{^{\prime}}\right) \cdot I(x+{x}^{^{\prime}}, y+{y}^{^{\prime}})}{\sqrt{{{\sum }_{{x}^{^{\prime}},{y}^{^{\prime}}}T\left({x}^{^{\prime}},{y}^{^{\prime}}\right)}^{2}\cdot {{\sum }_{{x}^{^{\prime}},{y}^{^{\prime}}}I(x+{x}^{^{\prime}}, x+{y}^{^{\prime}})}^{2}}}$$

The method $matchTemplate()$ in the $OpenCV$ library was used to compare the template image with the input images. The external libraries $cv2, numpy, glob$ and $workbook$ were also used. The detected microparticles are marked and corresponding coordinates are stored. Individual pearls are marked in the image using $imwrite()$ method. The coordinates obtained are combined with the value of the radius of the pearl, which is then used to identify the pearl chain. After the function finishes the comparison, the best matches can be found as global maximums $(TM\_CCORR\_NORMED)$ using the $minMaxLoc$ function. In case of a color image, template summation in the numerator and each sum in the denominator is done for all the channels. The result will still be a single-channel image, which is easier to analyze. The center coordinate of each microparticle is extracted from the coordinate data set. Each coordinate is used to search for the adjacent microparticle using the condition $2r+c$. All the microparticles nearest to a chain are identified and grouped to a dataset, duplicates are removed, and pearl chains are categorized into pearl chain count, ${C}_{L}$ where $L=\left[\mathrm{2,3},4\dots 18\right]$. Identified pearl chains are stored in a list, which at the end of processing the pearl chain length and count is stored in an excel sheet. The precision of image detection can be controlled by changing the values of the threshold in the code. Method $excelWrite()$ is used for representing the data of bulk image processed in excel sheet.
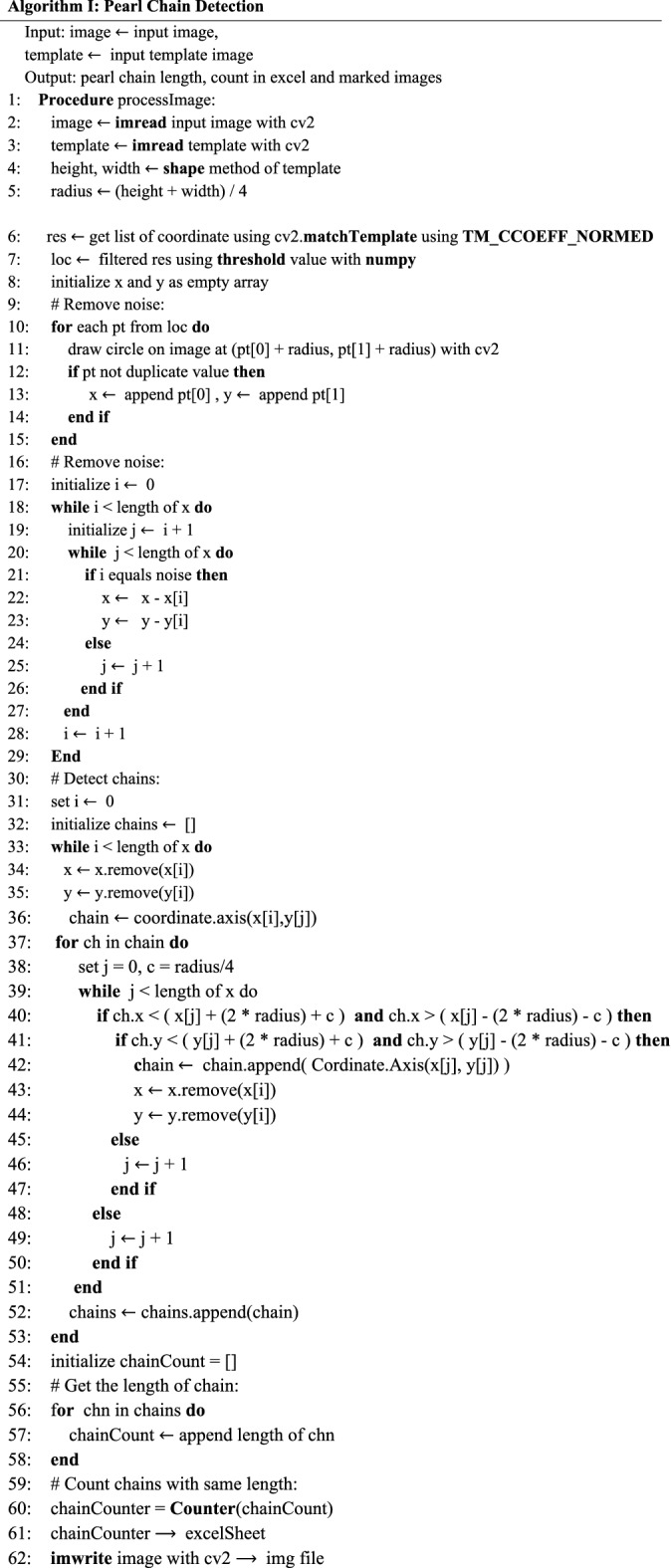


#### Machine learning models trainings

Prediction of target variable (applied voltage) was done using 18 predictors—which are the pearl chain features. Each predictor represents the number of particles in a pearl chain. Predictors of our model were extracted from the micrographs (Fig. 2). The value of each predictor is the number of microparticles in a pearl chain at an applied voltage. Pearl chain count, ${C}_{L}$ where $L=\left[\mathrm{2,3},4\dots 18\right]$ represents the number of pearl chains of a specific chain length $L$, ${C}_{L}$ values for all images taken at different voltages are stored in as a matrix and used as features or predictors in order to represent the DEP force. The pearl chain formations were recorded at different voltages. We hypothesize that pearl chain counts ${C}_{L}$ in a micrograph at different voltages ${V}_{x}$ where $1\le x\le 10$, is a thorough representation of DEP micrographs. Evident from the micrographs, pearl chain formations were observed at voltages as low as 2 V. However almost all the pearl chains had not more than 2 microparticles (${C}_{2}$). At 3 V, 84% of the pearl chains were ${C}_{2}$ and 15% of them were ${C}_{3}$. For voltages beyond 5 V, ~ 40% of the pearl chains have more than 4 microparticles $({C}_{4})$. Above 7 V, ${C}_{8}-{C}_{10}$ is significant (6.7%). In the 7–10 V range, ${C}_{2}-{C}_{5}$ percentages were very low and majority of the pearl chains had more than 8 microparticles ${C}_{8}$.

ML analyses were performed using the Orange toolbox by writing Python scripts accessing the Orange API. Additional functionalities like feature importance were developed using Python Script widgets. 80% of dataset is assigned as training data set and 20% to testing. Missing values were replaced with the median value of the features. Features with higher dominance in predicting the targets are identified from training sets. As shown in Fig. S1, different ML architectures such as K-Nearest Neighbor (KNN), Support Vector Machine (SVM), Random Forest, Neural Networks, and Linear Regression were trained on the dataset extracted from the PS microbeads micrographs. The Python scripts used for the machine learning is made available via this Github Link (https://github.com/skmidhun09/image_detection_python).

#### Feature importance estimation for maximum relevance and minimum redundancys

**Figure 3:**
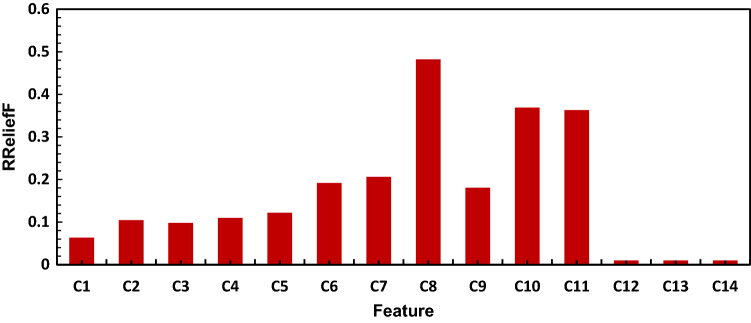
Ranking the importance of the predictors using $RReliefF$ algorithm to find a subgroup of high-prevalence features.

Extraneous features degrade the performance of a model while also increase computing costs. It is critical to find a subgroup of high-prevalence features. Some of the features have a considerable impact on the response model than others. We ranked the importance of features or predictors using $RReliefF$ algorithm with k-nearest neighbors. $RReliefF$ is a function that works with continuous target. $RReliefF$ penalizes features who offer different values to neighbors with the same response values, and rewards features who give different values to neighbors with different response values. Figure 3 shows the features importance ranking obtained by implementing $RReliefF$. Among the ${C}_{L}$ predictors where $L=\left[\mathrm{2,3},4\dots 18\right]$, ${C}_{8}$ was found to be the most important feature with a weight of 0.48, followed by ${C}_{10}$ and ${C}_{11}$ with importance weight value of 0.37 and 0.36 respectively. ${C}_{1}$ had the least score of 0.06 and ${C}_{12}-{C}_{14}$ were found to be insignificant.

### Convolutional neural networks as base architecture for deep regression

Convolutional Neural Networks (CNN) was used to extract local trends from spatio-temporal patterns of pearl chain formation. CNNs have at least one layer that uses the convolution operation to extract features^57,58^. CNNs are used in image processing applications, including automated histopathological image segmentation^59^, automated reconstruction of low-contrast image such as magnetic resonance imaging (MRI)^60,61^, quantify cyanobacteria from hyperspectral images^62^, medical image processing for direct disease diagnosis^63,64^, as well as in other disciplines including speech recognition^58,65^ and weather forecast^57,66^.

**Figure 4:**
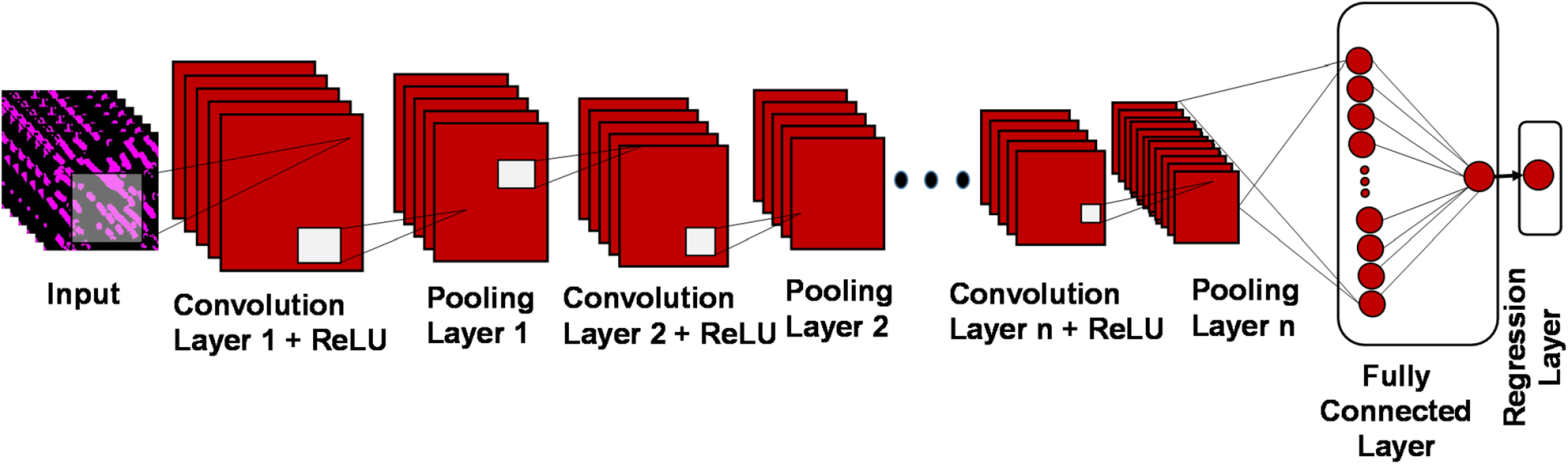
Modification of the Convolutional Neural Networks base architectures (viz: AlexNet, MobileNetV2, GoogLeNet and ResNet-50) from the conventional classification framework to a regression framework.

We have used four pre-trained CNN architectures viz*.* AlexNet^67^, MobileNetV2^68,69^, GoogLeNet^70^ and ResNet-50^71^ as the base architectures for deep regression analysis. Table 2 presents a brief overview of these pre-trained CNN architectures. All these architectures were initialized as pre-trained version of the networks which were initially trained on ImageNet dataset for classification. As illustrated in Fig. 4, the pre-trained CNN architectures consist of an input layer, which represents the pixel matrix of an input image, followed by a series of convolution layers that uses Rectified Linear Unit (ReLU) activation. Between two convolution layers is a pooling layer, where max pooling operation is done to down-sample the convoluted image (feature map). Subsequently a fully connected layer where all the inputs are connected along with softmax layers. In order to retrain these pre-trained networks for regression, we remove the last softmax layers from the base architectures (AlexNet, MobileNetV2, GoogLeNet and ResNet-50), employed in the context of classification, and then replace the final fully connected layer, the softmax layer, and the classification output layer with a fully connected layer of size 1 (the number of output variable) with linear activations and a regression layer. As a result, the last layer is a regression layer, whose output dimension corresponds to that of the target space.

**Table 2:** Brief overview of the pre-trained CNN architectures.

Architecture	Main Finding	Number of Parameters	Depth	Numbers of Layers	Input Size	Dataset	Error Rate
*AlexNet*	Uses Dropout and ReLU	61 million	8	25	227 × 227 × 3	ImageNet	16.4
*ResNet-50*	Resistant to overfitting, due to symmetry mapping-based skip linkages	25.6 million	50	177	224 × 224 × 3	ImageNet	3.57
*MobileNetV2*	Inverted residual framework	3.5 million	53	154	224 × 224 × 3	ImageNet	–
*GoogLeNet*	Block and concatenation concepts, varied filter size, increased depth	7 million	22	144	224 × 224 × 3	ImageNet	6.7

Notable hyperparameters such as the learning rate $\alpha$ and batch size ${n}_{b}$ were appropriately tuned to minimize the cost function and speedup optimization while ensuring the models converge to the global minimum, thereby solving the problem of overfitting^69,72^. Table 3 presents the CNN hyperparameters used. A series of adaptive learning rate algorithms have recently been developed, Adaptive Moments (Adam)^72^, root mean square propagation (RMSProp)^72,73^, and stochastic gradient descent with momentum (SGDM)^74^ optimizers explored in this work are among the most widely used optimization algorithms. Table 4 presents a concise overview of these algorithms.

**Table 3:** Hyperparameters used.

Hyperparameters	Value
Batch size (${n}_{b}$)	10
Learning rate ($\alpha$)	0.0001
Optimizers	RMSProp, SGDM, ADAM
Number of Epochs	100
Validation frequency	30
Execution environment	CPU

**Table 4:** Overview of optimization algorithms.

Optimizer	Update Rule	Description
RMSProp	${V}_{dW}= \beta {V}_{dW}+\left(1-\beta \right){dW}^{2}$ $W=W- \alpha \frac{dW}{\sqrt{{V}_{dW}+\varepsilon }}$	i. An extension of gradient descent called Adaptive Gradient, or AdaGrad ii. It avoids drastically lowering learning rates by converting the gradient accumulation to an exponentially weighted moving average iii. For that weight, RMSProp only considers recent gradients
ADAM	${S}_{dW}= {\beta }_{1}{S}_{dW}+\left(1-{\beta }_{1}\right)dW$ ${V}_{dW}= {\beta }_{2}{S}_{dW}+\left(1-{\beta }_{2}\right){dW}^{2}$ ${Scorr}_{dW}= \frac{{S}_{dW}}{{\left(1-{\beta }_{1}\right)}^{t}}$ ${Vcorr}_{dW}= \frac{{V}_{dW}}{{\left(1-{\beta }_{2}\right)}^{t}}$ $W=W- \alpha \frac{{Scorr}_{dW}}{\sqrt{{Vcorr}_{dW}+\varepsilon }}$	i. ADAM is an improvement to the RMSProp optimizer that incorporates momentum method ii. It is an algorithm for handling sparse gradients in noisy problems iii. ADAM is simple to set up, and the default settings work well for most problems
SGDM	${S}_{dW}=\beta {S}_{dW}+\left(1-\beta \right)dW$ $W=W- \alpha {S}_{dW}$	i. The SGDM approach aids in the acceleration of gradient vectors in the proper directions, resulting in faster convergence ii. It requires more training time and requires hyperparameter tuning than ADAM and RMSProp iii. It is more effective than traditional gradient descent

The image files collected from micrographs of pearl chain formation at various voltages ranging from 1 to 10 V were used to perform deep learning analysis.

#### Image preprocessing and segmentation

**Figure 5:**
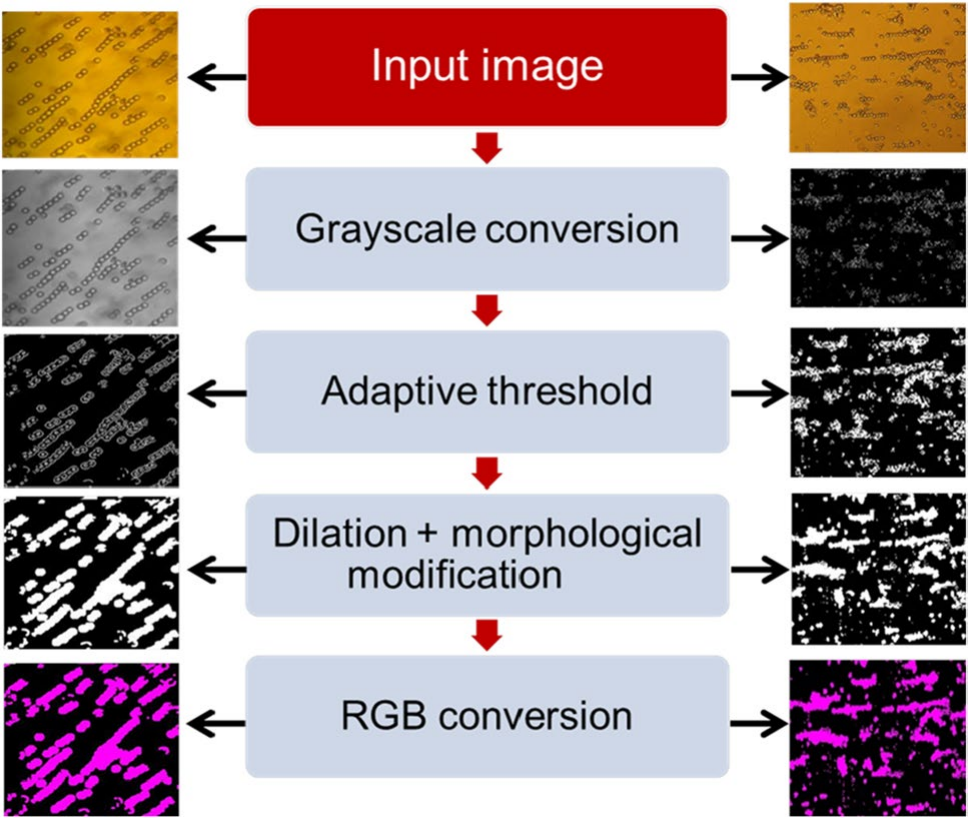
A flowchart of morphological operations carried out on the micrographs for image segmentation, the output segmented image $O(u, v)$ is generated by concatenating ${\mathrm{I}}^{\theta }\left(u,v\right)$ thrice to form the equivalent true color (RGB) image.

In order to improve the computational time and accuracy, we applied an optimum adaptive threshold method to reduce the complexity of pearl chain images^75^. Figure 5 depicts the flowchart of the image analysis and segmentation technique. Also, we summarized all of the steps of the segmentation process in Algorithm II.

The MATLAB Image Processing Toolbox was utilized to prototype the methods for image processing in this research. Grayscale conversion, adaptive thresholding segmentation, and morphological operations were all part of the image processing procedures were carried out systematically for 4000 images^5,76–78^. $I(u, v)$ are grayscale images of the pearl chains in a Euclidean space $E$. Through intensive thresholding of the pearl chain regions from the micrograph, adaptive global threshold was employed to accomplish segmentation of the pearl chains. The threshold value selected was obtained through image histogram to produce the binarized output ${\mathrm{I}}^{\beta }\left(u,v\right)$.

7 $$ {\text{I}}^{\beta } \left( {u,v} \right) = \left\{ {\begin{array}{*{20}l} 1 \hfill & {I\left( {u,v} \right) \ge \alpha } \hfill \\ 0 \hfill & {I\left( {u,v} \right) < \alpha } \hfill \\ \end{array} } \right. $$

$\alpha$ is the adaptive threshold value applied to original input image $I(u, v)$ to get the resultant image, denoted with ${\mathrm{I}}^{\beta }\left(u,v\right)$. In the next steps, a morphological operation called dilation is applied on ${\mathrm{I}}^{\beta }\left(u,v\right)$ using structuring element ${S}_{1}$ (an array of horizontal and vertical lines). The dilation of ${\mathrm{I}}^{\beta }\left(u,v\right)$ by ${S}_{1}$ is mathematically defined as in^76,77^ by Eq. (8) and (9) below, where ${\widehat{S}}_{1}$ is the translation of the array ${S}_{1}$ by the vector $z$ and $\varnothing$ is a null set:

8 $${\mathrm{I}}^{\gamma }\left(u,v\right)={I}^{\beta }\left(u,v\right) \oplus {S}_{1}$$

9 $${\mathrm{I}}^{\gamma }\left(u,v\right)=\left\{z\in E|{\left({\widehat{S}}_{1}\right)}_{z}\cap {\mathrm{I}}^{\beta }\left(u,v\right)\ne \varnothing \right\}$$

Now, performing a morphological closing operation defined in^77,78^ as the erosion of ${\mathrm{I}}^{\gamma }\left(u,v\right)$ by a horizontal structuring element ${S}_{2}$, followed by dilation of the resulting image by ${S}_{2}$, we obtain ${\mathrm{I}}^{\delta }\left(u,v\right)$ as shown below:

10 $${\mathrm{I}}^{\delta }\left(u,v\right)= {\mathrm{I}}^{\gamma }\left(u,v\right)\circ {S}_{2}$$

11 $${\mathrm{I}}^{\delta }\left(u,v\right)=\left\{z\in E|{\left({\widehat{S}}_{2}\right)}_{z}\cap \left(\left\{z\in E| {\widehat{S}}_{2}\subseteq {\mathrm{I}}^{\gamma }\left(u,v\right)\right\}\ne \varnothing \right)\right\}$$

Then, we fill the holes of the pearl chains and cleared its border. Then taking a morphological closing operation defined in^77^ as the dilation of ${\mathrm{I}}^{\delta }\left(u,v\right)$ by a horizontal structuring element ${S}_{2}$, followed by erosion of the resulting image by ${S}_{2}$, we obtain as shown below:

12 $${\mathrm{I}}^{\theta }\left(u,v\right)= {\mathrm{I}}^{\delta }\left(u,v\right) \cdot {S}_{2}$$

13 $${\mathrm{I}}^{\theta }\left(u,v\right)=\left\{z\in E| {\widehat{S}}_{2}\subseteq \left(\left\{z\in E|{\left({\widehat{S}}_{1}\right)}_{z}\cap {\mathrm{I}}^{\delta }\left(u,v\right)\ne \varnothing \right\}\right)\right\}$$

In the final step, the output segmented image $O(u, v)$ is generated by concatenating ${\mathrm{I}}^{\theta }\left(u,v\right)$ thrice to form the equivalent true color (RGB) image.

14 $$O\left(u, v\right)={\mathrm{I}}^{\theta }\left(u,v\right) \cap {\mathrm{I}}^{\theta }\left(u,v\right)  \cap {\mathrm{I}}^{\theta }\left(u,v\right)$$

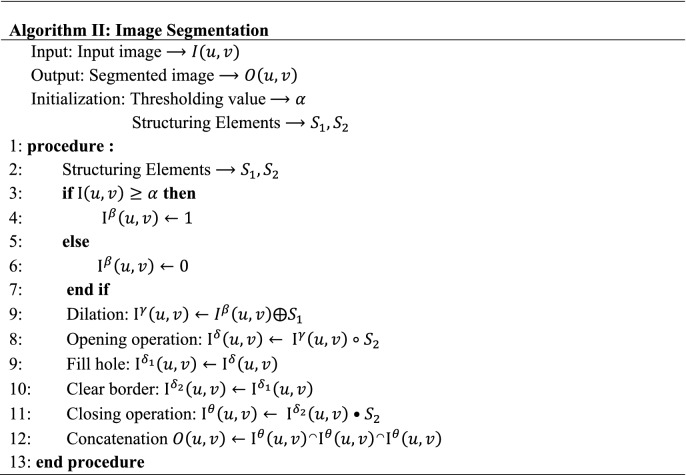


The MATLAB code used for the image processing task described above can be accessed through this Github link (https://github.com/AjalaSunday/Neural-Networks-Fall-2021/blob/16ac1708307431272fc2c5c72ee25594d0a81446/PreprocessingPCFImages.m).

## Results and discussion

### Model testing and evaluation metrics

The models were assessed for their performance by testing if pearl chain arrangements in a micrographs can be correlated to input voltages to find the model with the best performance using these four key performance metrics: Mean Absolute Error (MAE), Mean Relative Error (MRE), Mean Squared Error (MSE), R-squared, and Root Mean Square Error (RMSE)^48,49,57,79^. They are mathematically expressed as given by Eqs. (15–20) where $y$, $\widehat{y}$, and $\overline{y }$ define the actual value, predicted value, and mean of the $y$ values and $n$ is the number of samples:

15 $$\mathrm{MAE}= \frac{1}{n}\sum_{i}^{n}\left|{y}_{i}-{\widehat{y}}_{i}\right|$$

16 $$\mathrm{MRE}= \frac{1}{n}\sum_{i}^{n}\frac{\left|{y}_{i}-{\widehat{y}}_{i}\right|}{{y}_{i}}$$

17 $$\mathrm{MSE}= \frac{1}{n}\sum_{i}^{n}{\left({y}_{i}-{\widehat{y}}_{i}\right)}^{2}$$

18 $$\mathrm{R}-\mathrm{squared}=1-\frac{\sum_{i}^{n}{\left({y}_{i}-{\widehat{y}}_{i}\right)}^{2}}{\sum_{i}^{n}{\left({y}_{i}-\overline{y }\right)}^{2}}$$

19 $$\mathrm{RMSE}=\sqrt{ \frac{1}{n}\sum_{i}^{n}{\left({y}_{i}-{\widehat{y}}_{i}\right)}^{2}}$$

The prediction accuracy of the models can be defined is given by Eq. (20):

20 $$Accuracy= \frac{\sum_{i}^{n}\left|{y}_{i}-{\widehat{y}}_{i}\right|<0.5}{n}$$

The MAE assesses the average magnitude of errors in a group of predictions without taking into account their direction. It assesses the precision of continuous variables. MSE is often referred to as quadratic loss since the penalty is related to the square of the error rather than the error itself. When the error is squared, the outliers are given more weight, resulting in a smooth gradient for small errors. With an increase in error, MSE grows exponentially. The MSE value of a good model should be close to zero. RMSE is computed by taking the square root of MSE. RMSE is the more easily interpreted as it has the same units as the quantity. MAE, MSE, and RMSE can range from 0 to ∞. The goodness of fit of a regression model is represented by a statistical measure called R-squared. The optimal R-squared value is 1. The closer the R-square value is to 1, the better the model fits.

### Deep regression model for dielectrophoretic force estimation

**Figure 6:**
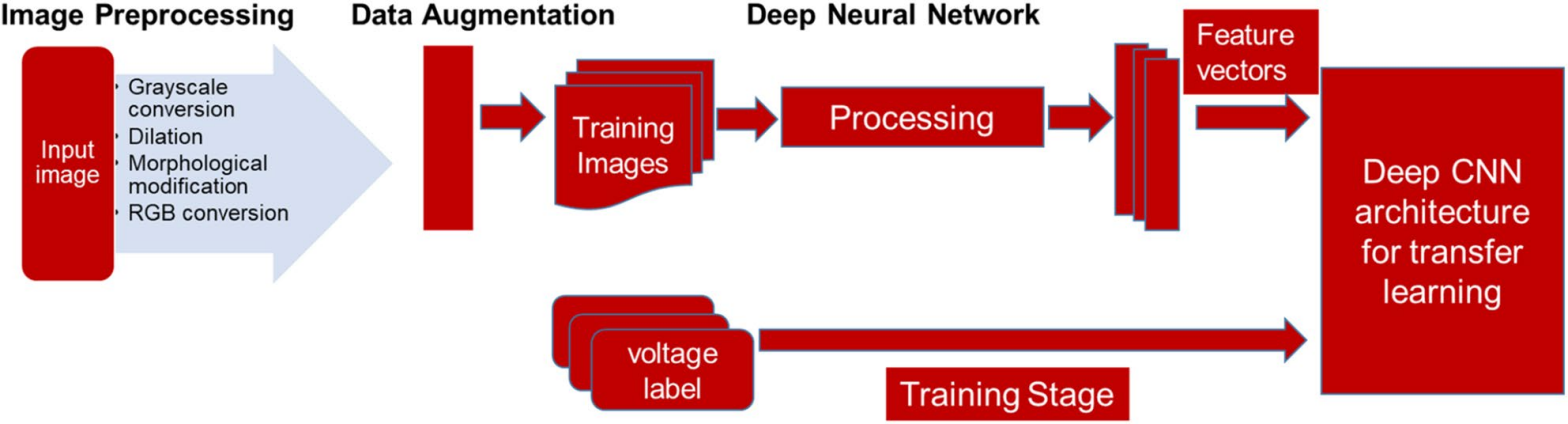
System overview showing the training phase of the models, the input image datasets were partitioned into 80% for the training and 20% for validation or testing.

After image processing and segmentation steps, the images are resized to fit the input layer dimension for each model (Table 2). These segmented image datasets are then augmented using augmentation procedures, such as randomly flipping them along the vertical axis and randomly translating them horizontally and vertically up to 30 pixels for training and validating the deep regression models (Fig. 6). Data augmentation keeps the networks from overfitting and ensure they adequately generalized. AlexNet, ResNet-50, MobileNetV2, and GoogLeNet were the four CNN architectures examined in this study. During the training phase of the models, the image datasets (2000 image samples each for yeast cells and PS microbeads) were partitioned into 80% (i.e. 1600 image samples each for yeast cells and PS microbeads) for the training and 20% (i.e. 400 image samples each for yeast cells and PS microbeads) for validation. The training was done in MATLAB R2021a, and the deep learning experiments were done with its Deep Learning toolbox. A DELL laptop with a five-core Intel 8th Generation processor served as our development system. The MATLAB code used for the deep learning described above is made available in this Githublink (https://github.com/AjalaSunday/Neural-Networks-Fall2021/blob/16ac1708307431272fc2c5c72ee25594d0a81446/RegressionCode.m).

**Figure 7:**
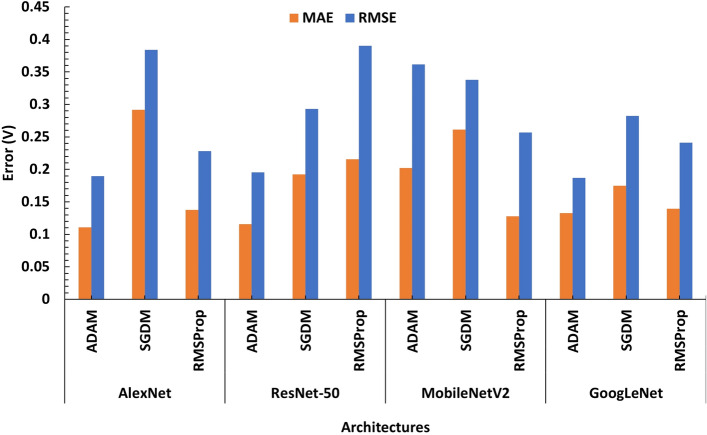
Model performance on yeast cells dataset for various architectures ResNet-50 with RMSPROP optimizer has the best validation RMSE of 0.0918 on test dataset, followed by the same ResNet-50 but with ADAM optimizer having a validation RMSE of 0.1241.

**Figure 8:**
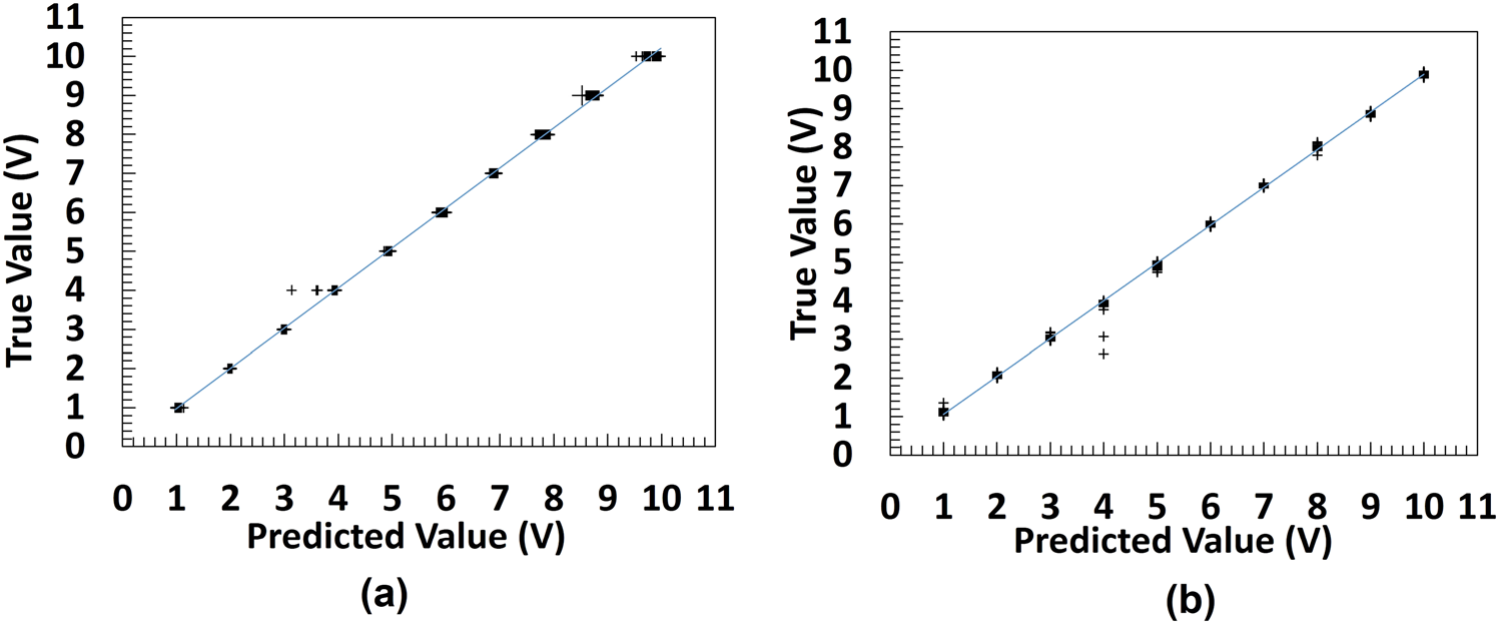
Line of best fit of the best deep regression models (**a**) ResNet-50 with RMSProp (**b**) ResNet-50 with ADAM.

By obtaining the R-squared, MAE, MRE, MSE, and RMSE values for the testing dataset base on the accuracy criteria, evaluation of the four architectures is performed. Tables 5 and 6 show the results achieved by the architectures and various optimizers in our experiments with yeast cells and microbeads respectively. As it can be seen in Table 5, all the models and optimization algorithms performed well above 95% based on the accuracy metric (also see Table S2). However, training the models on yeast cells dataset, ResNet-50 with RMSProp optimizer has the best validation RMSE of 0.0918 on test dataset, followed by the same ResNet-50 but with ADAM optimizer having a validation RMSE of 0.1241. This is also illustrated with the chart in Fig. 7. Figures S2 and S3 show the evolution of the validation RMSE for ResNet-50 with RMSProp optimizer and ResNet-50 with ADAM optimizer on the yeast cell dataset respectively while the regression lines are illustrated in Fig. 8a and b respectively.

**Table 5:** CNN deep regression model performance on yeast cells for various architectures

Architecture	Optimizer	MAE	MSE	RMSE	R-squared	Rank
AlexNet	ADAM	0.2089	0.0390	0.1976	0.995	8th
	SGDM	0.2009	0.0616	0.2483	0.993	11th
	RMSProp	0.1386	0.0403	0.2008	0.996	9th
ResNet-50	**ADAM**	**0.0864**	**0.0154**	**0.1241**	**0.999**	**2nd**
	SGDM	0.1609	0.0477	0.2185	0.994	10th
	**RMSProp**	**0.0661**	**0.0084**	**0.0918**	**0.999**	**1st**
MobileNetV2	ADAM	0.1072	0.0195	0.1398	0.999	4th
	SGDM	0.2099	0.0699	0.2645	0.993	12th
	RMSProp	0.1049	0.0216	0.1470	0.999	5th
GoogLeNet	ADAM	0.0781	0.0151	0.1230	0.999	3rd
	SGDM	0.1319	0.0290	0.1701	0.997	6th
	RMSProp	0.1038	0.0312	0.1768	0.997	7th

Significant values are in bold.

**Table 6:** CNN Deep Regression Model Performance on microbeads for various Architectures.

Architecture	Optimizer	MAE	MSE	RMSE	R-squared	Rank
AlexNet	**ADAM**	**0.1109**	**0.0305**	**0.1745**	**0.996**	**1st**
	SGDM	0.2919	0.1474	0.3839	0.985	11th
	RMSProp	0.1376	0.0520	0.2281	0.994	4th
ResNet-50	**ADAM**	**0.1329**	**0.0349**	**0.1869**	**0.998**	**2nd**
	SGDM	0.1924	0.0859	0.2931	0.99	8th
	RMSProp	0.2156	0.1524	0.3904	0.987	12th
MobileNetV2	ADAM	0.2022	0.1307	0.3616	0.99	10th
	SGDM	0.2614	0.1142	0.3379	0.99	9th
	RMSProp	0.1277	0.0660	0.2570	0.994	6th
GoogLeNet	ADAM	0.1159	0.0383	0.1956	0.995	3rd
	SGDM	0.1750	0.0798	0.2825	0.992	7th
	RMSProp	0.1395	0.0580	0.2409	0.993	5th

Significant values are in bold.

**Figure 9:**
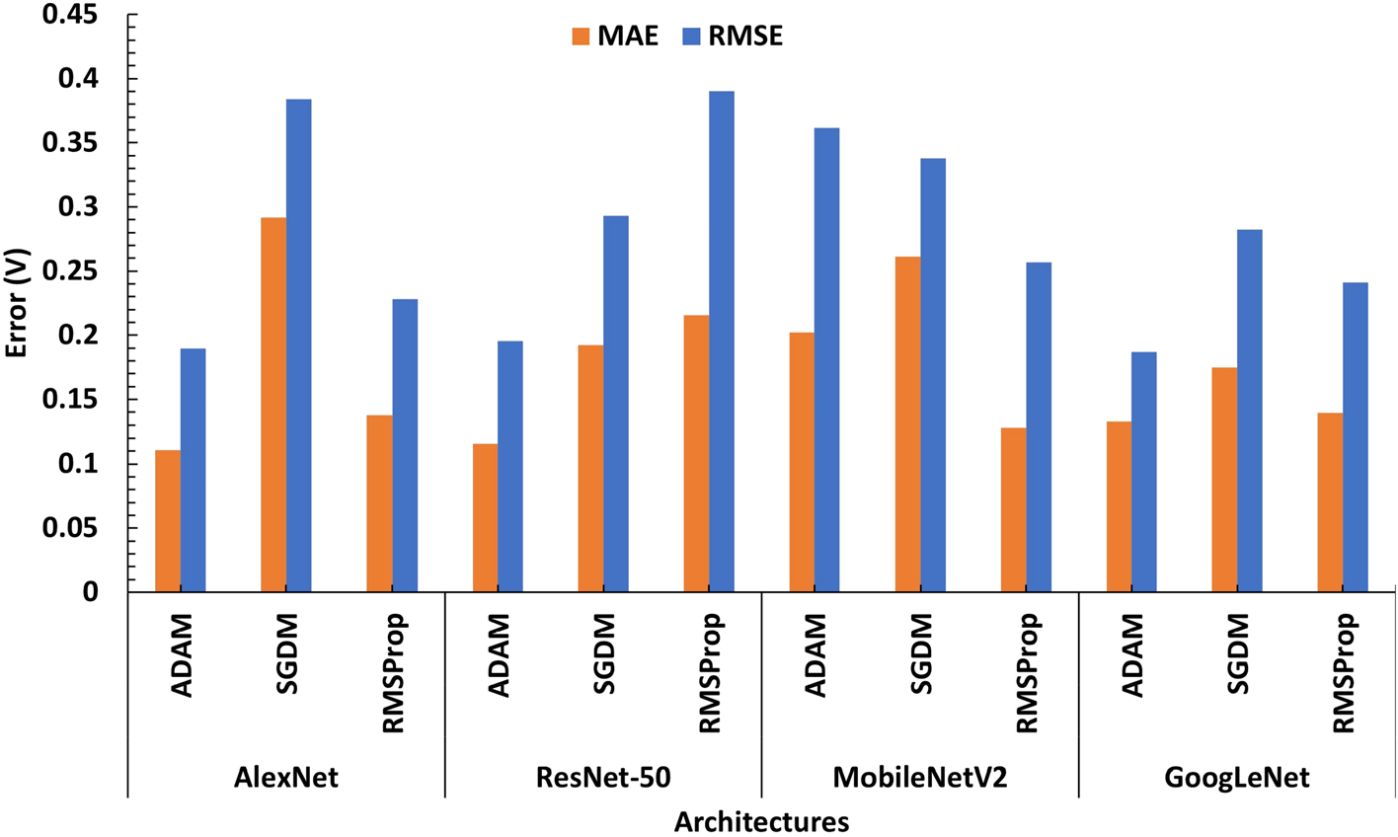
Model performance on microbeads dataset for various architectures AlexNet with ADAM optimizer has the best validation RMSE of 0.1745 on test dataset, followed by the same ResNet-50 but with ADAM optimizer having a validation RMSE of 0.1869.

**Figure 10:**
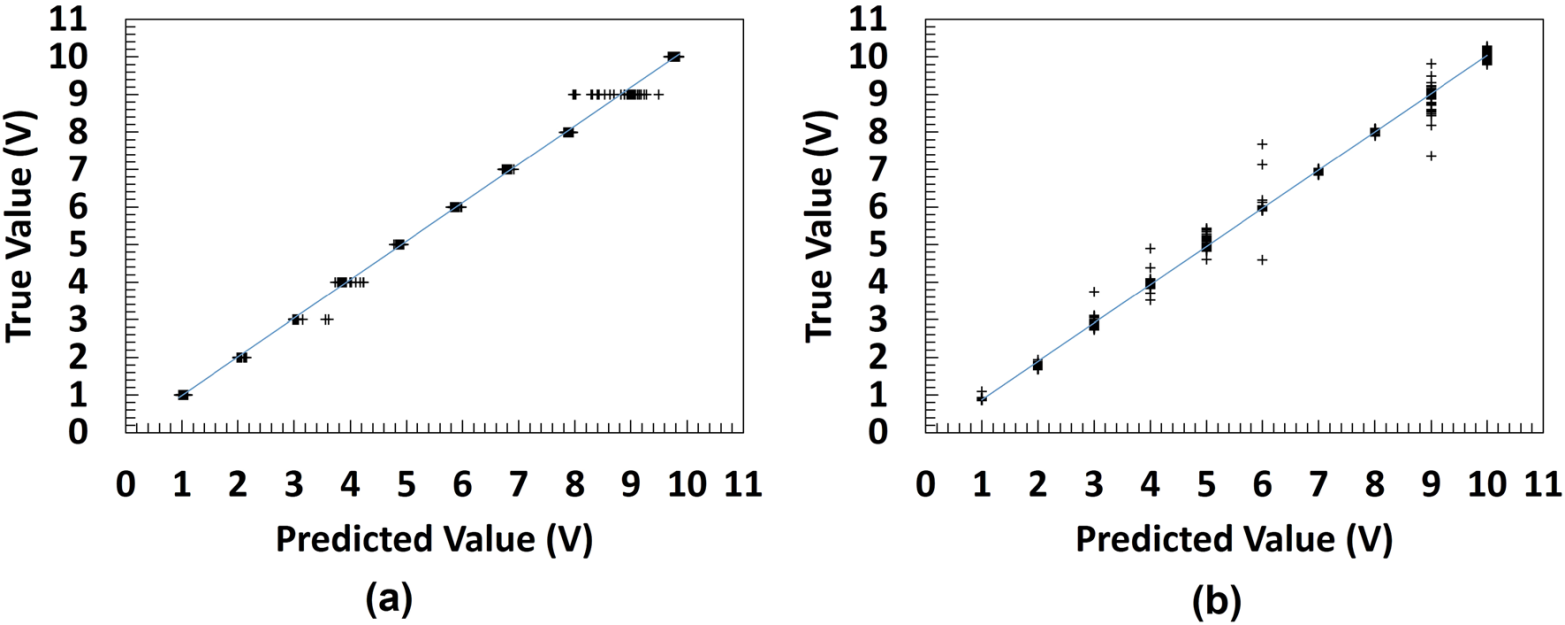
Line of best fit of the best deep regression models (**a**) AlexNet with ADAM (**b**) ResNet-50 with ADAM.

**Figure 11:**
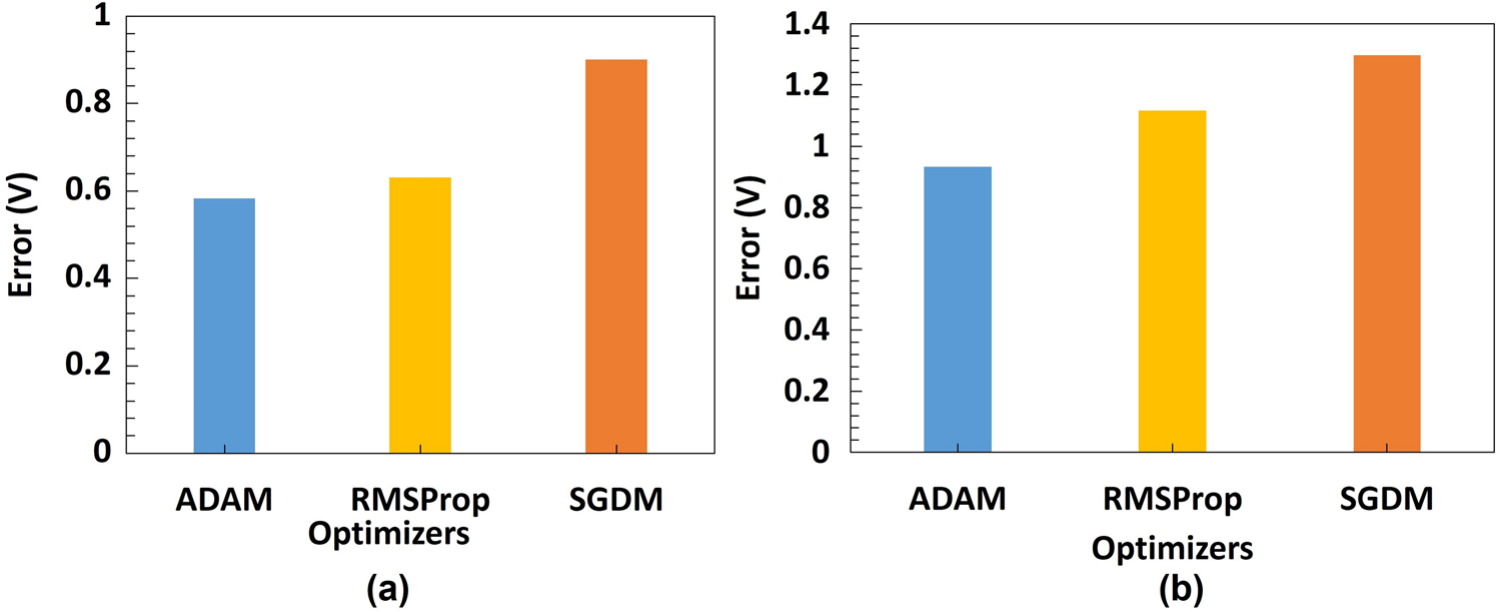
Sum of RMSE by Optimizer on (**a**) Yeast Cell Dataset (**b**) PS Microbead Dataset.

On the microbeads dataset, as it can be seen in Table 6, all the models and optimization algorithms performed well above 90% based on the accuracy metric (also see Table S3 and S4). AlexNet with ADAM optimizer have the best validation RMSE of 0.1745 across all models on the PS microbeads dataset followed by ResNet-50 also with ADAM optimizer with validation RMSE 0.1869. This is also illustrated with the chart in Fig. 9. Figures S4 and S5 show the evolution of the validation RMSE for AlexNet with ADAM optimizer and ResNet-50 with ADAM optimizer on the PS microbeads dataset respectively while the regression lines are illustrated in Fig. 10a and b respectively. A look at the performances of adaptive learning rate optimization algorithms explored in this work, we found that ADAM has the least sum followed by RMSProp and then SGDM come last, having the highest sum of RMSE on both datasets as shown in Fig. 11a and b.

## Conclusion

This paper presents an intelligent sensing framework capable of direct estimation of DEP force from pearl chain alignment of microparticles. We have tested the proposed models in an electrode-based dielectrophoretic system. The proposed deep regression models were extensively examined, and results were compared with conventional machine learning approaches. The intrinsic features of microparticle alignment like pearl chain length and count were extracted using image segmentation algorithms and used to generate training datasets. The results from the experiments show that the performance of the DL models proved to be optimal in terms of prediction accuracy and generalization ability compared to the ML models. ResNet-50 with RMSPROP gave the best performance, with a validation RMSE of 0.0918 on yeast cells while AlexNet with ADAM optimizer gave the best performance, with a validation RMSE of 0.1745 on microbeads. The regression model we developed can be extended to biosensing systems in order to estimate the variations in dielectric properties of microparticles.

## Supplementary Information

Supplementary Information.

## Data Availability

The dataset used for the current study is available on Dryad via this link. This is available to the reviewers but will be made available to the public after this article has been peered reviewed or upon request from the corresponding author.
