# Supplementary material for: Comparing machine learning and deep learning regression frameworks for accurate prediction of dielectrophoretic force
Source: Sci Rep. 2022 Jul 13;12:11971. doi: 10.1038/s41598-022-16114-5 (PMC9279499; doi:10.1038/s41598-022-16114-5)
Supplement: Supplementary file 1 — Supplementary Information. [file 41598_2022_16114_MOESM1_ESM.pdf]

Supplementary Material

Machine Learning Based Pearl Chain Detection

Input:

Super set-(Tr_i) = {Tr_1, Tr_2…. Tr_{10}} Where Tr = Training Sets (i =1 to 10)

Super set (Te_i) = {Te_1, Te_2…. Te_{10}} Where Te = Test Sets (i=1 to 10)

Machine Learning Algorithms:

LR, RF, KNN, SVM, NN (ML_i)

Where:

LR-Linear Regression
RF-Random Forest
KNN-Knowledge nearest neighbor
SVM – Support vector machine
NN-Neural Network

Output:

1, Best Approach Algorithm
2, Dominance in Feature Extraction
3, Predicted Values in best approach algorithms.

BEGIN
    FETCH the datasets from source
    PREPROCESS the training & test datasets (DS_i) as Tr_i, Te_i
    CONVERT Categorical values (Ci) into Numerical values (N_i)
    FOR i =1 to 10 DO
        CHOOSE the target (Ti) variable in Tr_i
        CHOOSE the Original (Or_i) variable in Te_i
        FEED Features of (Tr_i) for FEATURE EXTRACTION (Fe_i)
        IDENTIFY the Fe_i values with Greater Probability to cover maximum Fe_ivalues
        CHOOSE the Higher Dominance of Fe_i from Tr_i
    END FOR
    FOR *j*=1 to 5 DO
        FEED Fe_j into Machine learning Algorithms (ML_j)
        FIND the Predicted values (P_j) from ML_j
    END FOR
    FOR i =1 to 10 DO
        IF Or_i== P_i THEN
            $A_i = 0$
            A_i(Accuracy)= A_i + 1
        ELSE IFOr_i > P_i|| Or_i< P_i THEN
            A_i(Accuracy)= A_i - 1
        END IF
    END FOR
    FOUND ACCURACY (A_1, A_2…. A_5)
    FOR i = 1 to 10 DO
        Gr_i=A_i
        IF A_i > Gr_i THEN
            Gr_i=A_i
        EVALUATE greater (Gr_i) for the best Machine Learning algorithms
        END IF
    END FOR
END

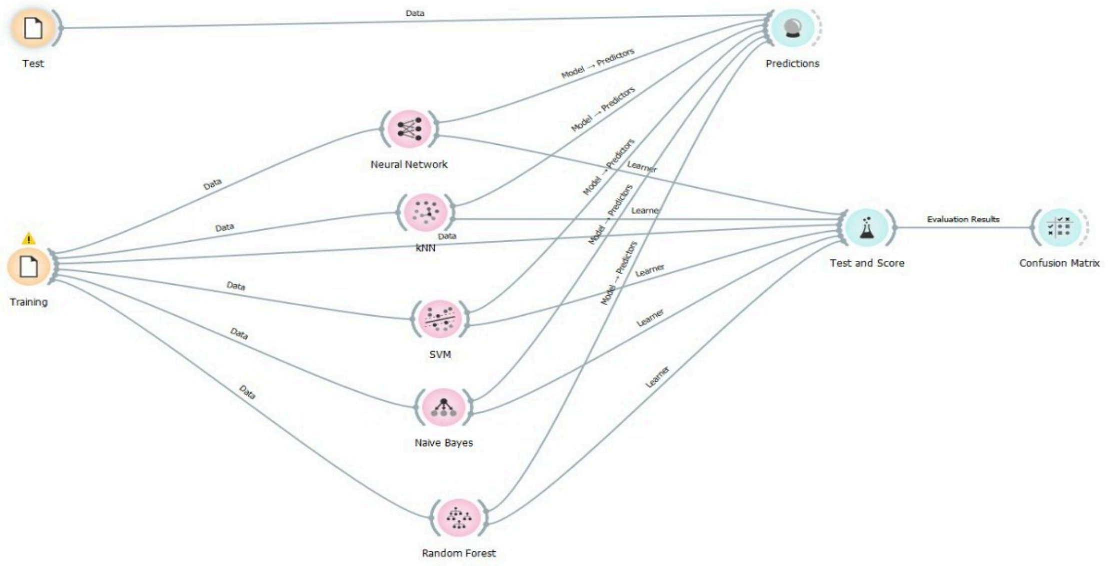

Fig. S1. Applying different machine learning algorithms on the pearl chain dataset using Orange

Input / Output

As input for datasets, we have a training set and a test set (Tr_i & Te_i), respectively. The prediction results are based on five machine learning algorithms. It finds dominant values in feature extracted, best approach algorithms among machine learning algorithms, and the best way to predict the values of test sets.

Preprocessing and Higher Dominance

Initially fetch the training and test datasets from the source. Preprocess the dataset by converting categorical values (Ci) into numerical values (Ni). Identify the target feature (Ti) from the training sets (Tr_i). Find the original predicting feature (Or_i) from the test sets (Te_i). Use the training sets (Tr_i) of feature extraction (Fe_i). Identify the Fe_i values with Greater probability to cover maximum features values. Finally, find the higher dominance features (Fe_i) from the training sets (Tr_i).

Accuracy Findings

Feed the higher dominance feature extracted values (Fe_j) into 5 Machine learning algorithms (ML_j). Comparing the accuracy of all algorithms based on the original value (Or_i) present in the test set and the predicted values (P_j) found using all algorithms (ML_j). Analyze and choose the most accurate machine learning algorithms based on the datasets collected from the source.

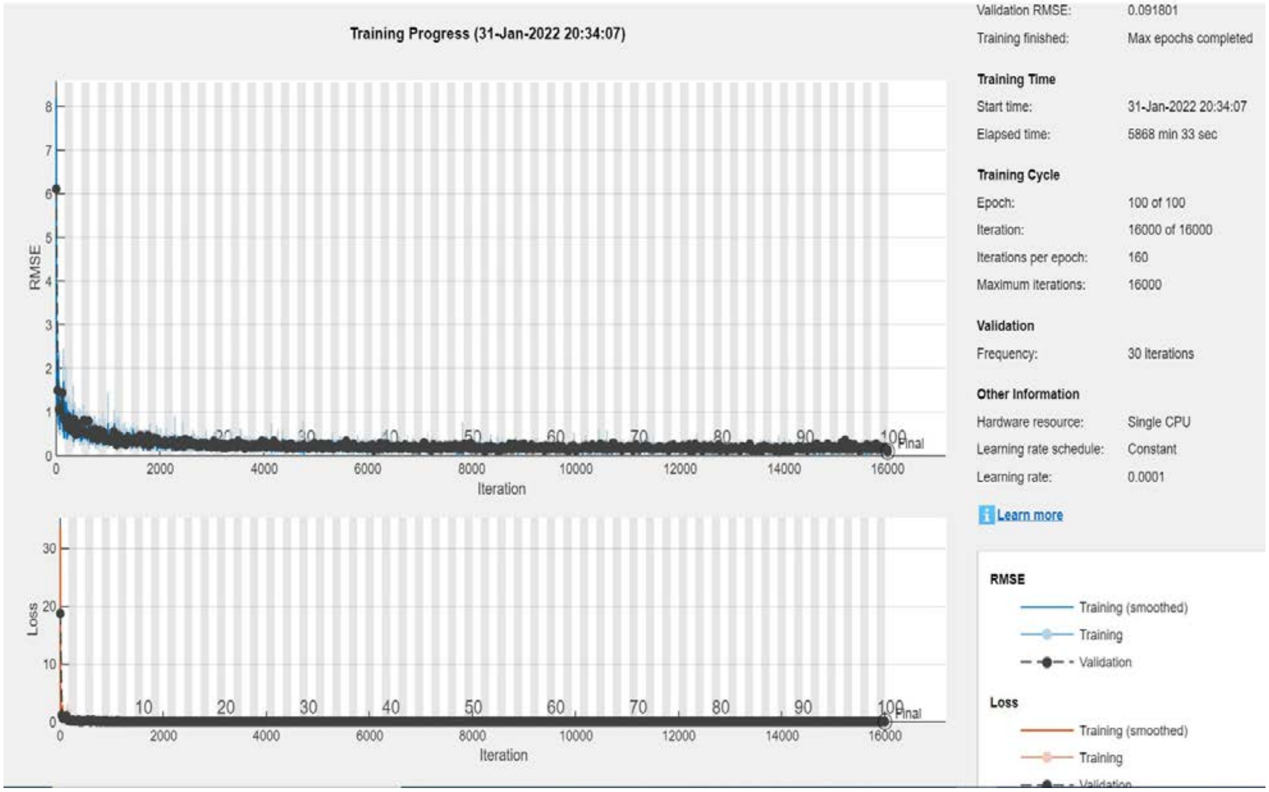

Fig. S2. Training Progress shows the Validation RMSE of AlexNet with ADAM

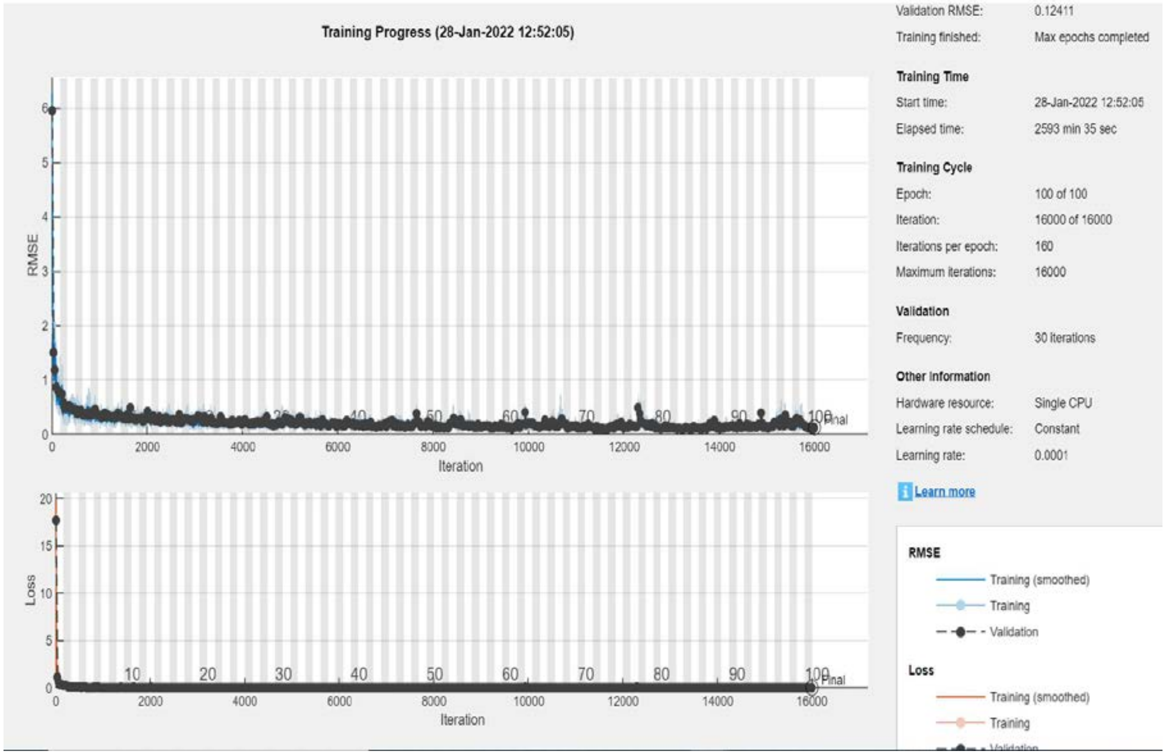

Fig. S3. Training Progress shows the Validation RMSE of ResNet-50 with RMSProp

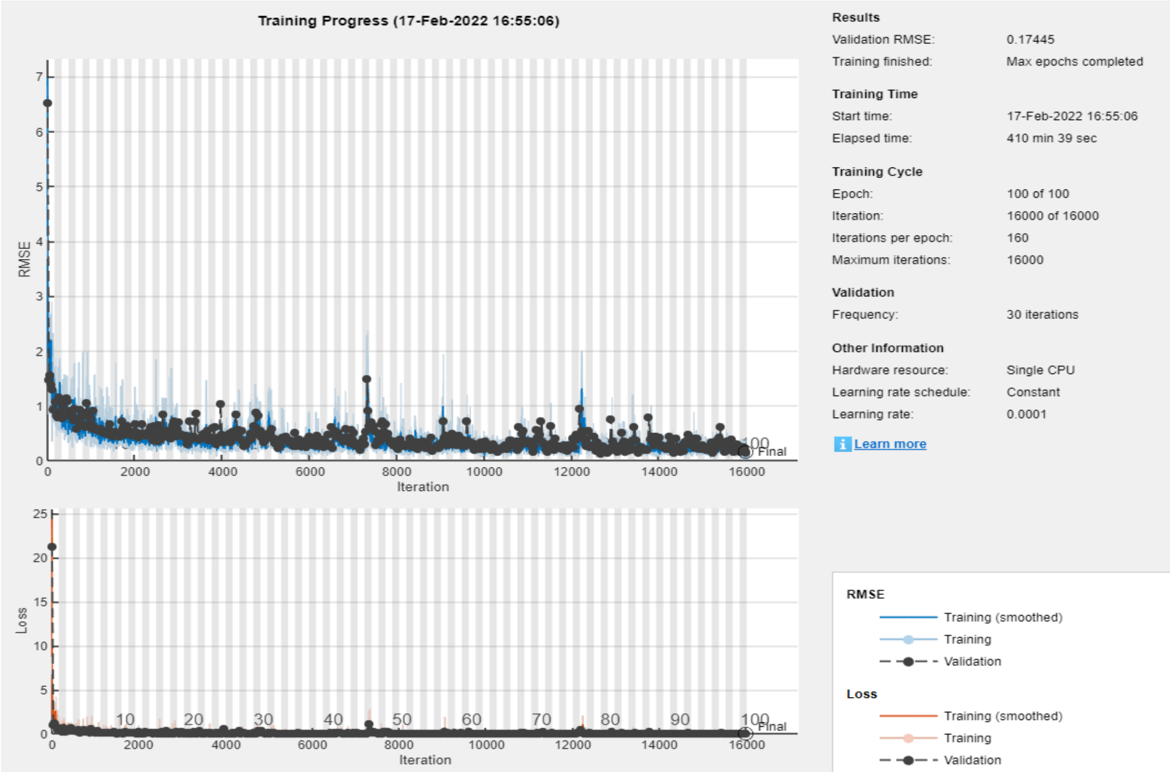

Fig. S4. Training Progress shows the Validation RMSE of AlexNet with ADAM

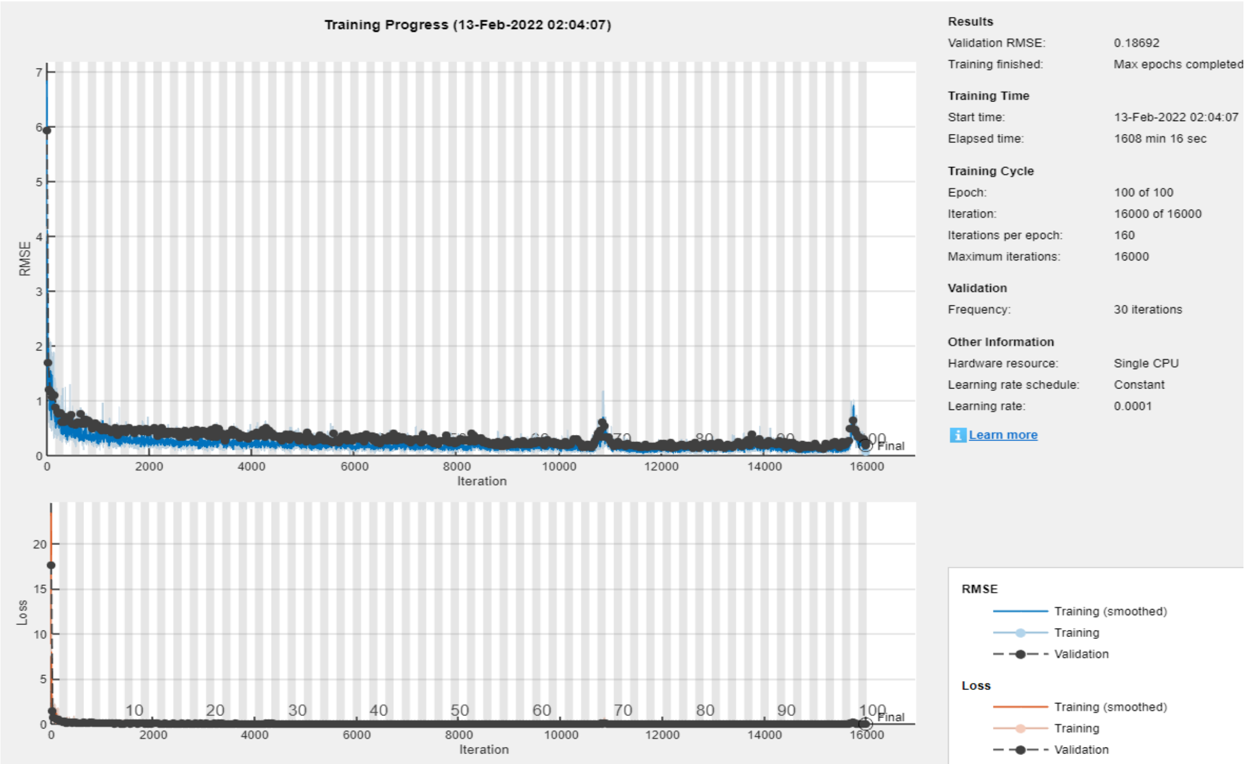

Fig. S5. Training Progress shows the Validation RMSE of ResNet-50 with ADAM optimizer

Table S1. CNN Deep Regression Model Performance on Yeast Cells for various Architectures

Architecture	Optimizer	Accuracy	MRE
AlexNet	ADAM	0.9550	0.0551
	SGDM	0.9575	0.0541
	RMSProp	0.9750	0.0191
ResNet-50	ADAM	0.9925	0.0210
	SGDM	0.9825	0.0452
	RMSProp	0.9925	0.0168
MobileNetV2	ADAM	0.9925	0.0255
	SGDM	0.9525	0.0562
	RMSProp	0.9975	0.0187
GoogLeNet	ADAM	0.9950	0.0247
	SGDM	0.9900	0.0306
	RMSProp	0.9925	0.0217

Table S2. CNN Deep Regression Model Performance on PS Microbeads for various Architectures.

Architecture	Optimizer	Accuracy	MRE
AlexNet	ADAM	0.9675	0.0257
	SGDM	0.8875	0.0803
	RMSProp	0.9750	0.0411
ResNet-50	ADAM	0.9600	0.0268
	SGDM	0.9200	0.0500
	RMSProp	0.9075	0.0379
MobileNetV2	ADAM	0.9075	0.0339
	SGDM	0.9125	0.0682
	RMSProp	0.9250	0.0204
GoogLeNet	ADAM	0.9750	0.0245
	SGDM	0.9225	0.0411
	RMSProp	0.9500	0.0230
